# Resting heart rate associations with violence exposure and posttraumatic stress symptoms: sex differences in children

**DOI:** 10.1186/s13293-024-00606-2

**Published:** 2024-03-28

**Authors:** Charis N. Wiltshire, Nicole Kouri, Cassandra P. Wanna, Sean T. Minton, John M. France, Mariam H. Reda, William Davie, Sattvik Basarkod, Sterling Winters, Rebecca Hinrichs, Anais F. Stenson, Tanja Jovanovic

**Affiliations:** 1 Department of Psychiatry and Neurosciences, Wayne State University School of Medicine, 3901 Chrysler Service Dr, Detroit, MI 48201 US; 2 Department of Psychiatry, Emory University School of Medicine, 69 Jesse Hill Jr Dr. SE, 30303 Atlanta, GA US

**Keywords:** Posttraumatic stress disorder, Child development, Adverse childhood experiences, Sex differences, Urban population, Heart rate

## Abstract

**Background:**

Traumatic events experienced in childhood can lead to increased risk of cardiovascular disorders in adulthood. Black Americans are disproportionately affected, as they are at increased risk for experiencing childhood trauma and cardiovascular diseases in adulthood. One of the hypothesized mechanisms of this association is through long-lasting dysregulation of the autonomic nervous system, a hallmark physiological biomarker of posttraumatic stress disorder (PTSD), which is twice as prevalent in women compared to men.

**Methods:**

Ninety-one, majority Black American children, aged 9 were recruited to be a part of our longitudinal study of child development at research centers in Atlanta, GA and Detroit, MI. Resting HR was measured through a electrocardiogram (ECG) recording using the Biopac MP150. Self-report measures of violence exposure and PTSD symptoms were administered by research staff.

**Results:**

Children with more violence exposure reported increased PTSS as well as lower resting HR. Regression analysis showed evidence of sex modifying this relationship, (B = -0.64, *p* < 0.05), such that the association between resting HR and PTSS was stronger in girls than in boys. In our exploratory analysis with standard clinical cutoffs of resting HR, the normative HR group was found to significantly moderate the relationship between violence exposure and PTSS in boys, (B = -2.14, *p* < 0.01), but not girls (B = -0.94, *p* = 0.27).

**Conclusion:**

In our sample of primarily Black urban children, we found that violence exposure was associated with slower, more adult-like HR, that girls showed greater PTSS associated with slower HR while boys did not, and that girls with lower than normative HR showed significantly higher PTSS compared to girls with normative HR. Our sample’s demonstration of psychological consequences in addition to the physiological implications could provide new information about a psychobiological sequelae of violence exposure.

**Supplementary Information:**

The online version contains supplementary material available at 10.1186/s13293-024-00606-2.

## Abstract (plain language summary)

Experiencing traumatic events in childhood can lead to increased risk of heart disease in adulthood. One of the ways this might happen is through long-lasting changes of the autonomic nervous system. This system is dysregulated in posttraumatic stress disorder (PTSD), which is twice as common in women compared to men. We explored whether resting heart rate (HR), a measure of autonomic functioning was associated with violence exposure in children, and whether this relationship was different in boys and girls. We also explored whether categorizing our sample into resting HR groups based off standardized norms for HR predicted differing relationships between violence exposure and posttraumatic stress symptoms (PTSS). Because childhood trauma and heart disease impact Black Americans at greater rates, we recruited our sample of 92 nine-year-old children from research centers in Atlanta, GA and Detroit, MI. We measured their resting HR, exposure to violence, and PTSS. We found that violence exposure was associated with lower HR overall, that girls showed greater PTSS associated with lower HR when compared to boys, and that boys with lower than normative HR showed a stronger association between violence exposure and PTSS compared to boys with normative HR. Future studies should examine potential mechanisms underlying this sex difference to best understand the long-term cardiovascular consequences for sex-related health disparities. Specifically, longitudinal studies may be able to help researchers understand how reduced HR during adolescents might lead to future cardiovascular disease and psychopathology.

**Supplementary Information:**

The online version contains supplementary material available at 10.1186/s13293-024-00606-2.

## Highlights

**•**We explored whether resting heart rate (HR), a measure of autonomic functioning, was associated with violence exposure, whether this relationship differs in boys and girls, and whether resting HR modified the relationship between violence exposure and posttraumatic stress symptoms (PTSS).
**•**In our sample of primarily urban children, we found that violence exposure was associated with lower HR, that girls showed greater PTSS associated with slower HR, and that boys with lower than normative HR showed a stronger association between violence exposure and PTSS compared to boys with normative HR.
**•**Our results shed light on the psychobiological sequelae of violence exposure.

**Supplementary Information:**

The online version contains supplementary material available at 10.1186/s13293-024-00606-2.

## Background and introduction

Exposure to violence and trauma in childhood have been linked with greater risk for cardiometabolic dysfunction and disorders later in life [1]. There is well-established literature demonstrating the associations between posttraumatic stress disorder (PTSD) and coronary heart disease and other physical health consequences such as health-related quality of life, musculoskeletal pain, cardio-respiratory symptoms, and gastrointestinal health [2–4]. Emerging scientific theories suggest that diseases experienced in adulthood such as cardiovascular disease and hypertension can be caused by developmental and biological disruptions in childhood [5]. Black Americans in particular suffer from cardiovascular diseases at disproportionally higher rates compared to White Americans [6]. Black children in the US are at greater risk of experiencing trauma or violence due to historic policies of structural racism and inequity [7–9]. While evidence indicating higher rates of trauma exposure in Black children compared to White children has been consistent [10], evidence suggesting elevated risk for PTSD in response to the higher rates of violence exposure in Black children has been mixed [11], potentially due to variable physiological responses to trauma.

One of the ways violence exposure can impact physiological mechanisms is by disrupting the normal functioning of the autonomic nervous system (ANS). Heart rate (HR) is influenced by the sympathetic nervous system in response to stress. Individuals with PTSD have shown increased sympathetic arousal through increased HR [12, 13]. The pioneering longitudinal work in this field has shown evidence to the association between trajectories of rate of HR decline in childhood and implications for higher cardiovascular risk in adulthood. They found that individuals with the highest decreasing resting HR trajectory were associated with cardiac mass and subsequent cardiovascular related risk [14]. In children, changes in HR have been associated with emotion dysregulation and varying psychopathologies [15–18]. In a recent longitudinal study, Haag et al. 2019 examined HR as a risk factor for posttraumatic stress symptoms (PTSS) in a sample of primarily White children immediately following trauma exposure [19]. They found that elevated mean HR during trauma narratives was associated with higher PTSS symptoms. However, in a sample of Black American children, decreased HR was associated with accelerated epigenetic aging in children with high rates of violence exposure [20]. Further, a recent study found lower resting HR to be a mediator between interpersonal violence and adverse psychopathology in children [21]. These somewhat contradictory findings indicate the need for more diverse samples to accurately depict the impact violence and trauma may have on psychopathology through cardiovascular mechanisms.

It is important to explore sex difference in these associations because pubertal timing plays an important role in physiological functioning of children and there are important sex differences in PTSD risk and rates [22–25]. A recent meta-analysis found evidence to suggest girls display greater mean HR compared to boys, an indication of differences in autonomic functioning [26]. Similarly, trauma has been found to accelerate pubertal timing in girls but not boys [27]. If average HR is to be explored as a mechanistic response to trauma or violence, sex differences could further contribute to differing risk for later psychopathology.

Population-based studies have found significant use of resting HR percentiles and cut offs to be good indicators of function, including systolic blood pressure, aerobic fitness, dehydration, obesity, and other known risk factors for cardiovascular disease [28–30]. Recently, resting HR cutoffs were found to be associated with cardiovascular risk factors in adolescents [31]. These findings support the use of resting HR cut offs as a biomarker for health and non-healthy cardiovascular functioning.

The current study examined sex differences in the relationship between violence exposure and resting HR and whether lower HR is a potential risk factor for negative mental health outcomes. We hypothesized that exposure to violence would be associated with lower resting HR in our sample of primarily Black, urban children. We also hypothesized that the association between HR and PTSD symptoms would be moderated by sex. In addition, we performed exploratory analysis with participants categorized into normative versus blunted resting cardiac functioning around CDC recommended standardized ranges of resting HR for 9-year-old children to see if lower resting HR, relative to normative reference points, was associated with negative mental health outcomes.

## Methods

### Participants

Child participants and their caregivers were recruited at age 9 years old (*M* = 9.4, *SD* = 0.37) from two sites, Detroit, MI and Atlanta, GA, through community recruitment, online advertisements, and study referrals. Caregivers that expressed interest in participating in research were contacted by study staff and scheduled for an appointment at our research center. During the study visit, caregivers provided informed written consent for themselves and their child to participate in the study. Child participants expressed verbal assent. All study protocols were approved by the Emory University Institutional Review Board and the Wayne State University Institutional Review Board.

Of the 102 participants recruited to participate in a three-year longitudinal child development study, 10 were excluded due to missing HR data and one was lost to follow-up with incomplete clinical assessments, leaving the final sample for analysis of 92 children (44 female). The ten individuals excluded from the analysis did not differ in age or sex, nor did they differ in levels of PTSS or violence exposure compared to the 92 children included in our analysis. The current analysis included only data from the baseline visit. Most participants self-identified as African American or Black (82.6%), with smaller numbers self-identifying as white or other (17.4%).

## Assessments

Rates of violence exposure were measured using the Violence Exposure Scale for Children-Revised (VEX-R) [32]. This validated instrument uses a 22-item cartoon-based self-report interview to assess the child’s lifetime exposure to violence. The VEX-R captures lifetime frequency of direct violence exposure and witnessed violence events. The total VEX-R scores used in our analysis represent the total number of different types of violence exposure both experienced and witnessed by the child. The University of California Los Angeles Post-Traumatic Stress Disorder Reaction Index for DSM-5 (UCLA-RI-5) was administered verbally by trained research staff to child participants to assess trauma-related PTSD symptoms [33]. The UCLA-RI-5 has been used extensively across a variety of trauma types, age ranges, settings, and cultures. The UCLA-RI-5 consists of 27 items assessing frequency of symptoms experienced within the last month using a five-point frequency scale (0 = None of the Time, 1 = Little of the time, 2 = Some of the time, 3 = Much of the time, 4 = Most of them time). Excellent internal reliability and test-retest reliability have been reported, and considerable data is available regarding the instrument’s validity [34]. Pubertal timing was captured through a self-report measure, the Pubertal Development Scale (PDS). The interview-based scale queries major physical changes during puberty through five questions relevant to each biological sex from which a mean score is calculated as a summary index of pubertal development [35]. 

Psychophysiological data were collected using Biopac MP160 for Windows (Biopac Systems, Inc. Aero Camino, CA). Heart Rate (HR) was continuously measured with Ag/AgCl electrodes in the Lead II placement of electrodes using the electrocardiogram (ECG) module. The acquired data were filtered, rectified, and smoothed using MindWare software (Mindware, Inc.) and exported for statistical analysis. Mindware identifies R-waves and R-R intervals (i.e., the time between heart beats), and detects artifacts, which were visually inspected and corrected. The ECG signal was sampled at 1000 Hz, amplified by a gain of 2000, filtered with a Hamming windowing function, and with a 60 Hz notch filter. Variables of interest were created from measurements acquired during the first 60 s of a fear conditioning task described in previous studies. For the purposes of our analysis and interpretation, resting HR was defined as the mean HR averaged over the first 60 s of a fear learning paradigm, before the introduction of any stimuli. Participant’s resting HR was compared to age appropriate normative ranges provided by the CDC and participants were stratified into “normative resting HR” or “lower-than-normal resting HR” groups [36]. The normative resting HR for children aged 9–11 years old is 83 beats per minute (BPM) for girls and 78 bpm for boys.

We also evaluated high-frequency heart rate variability (HF-HRV) of electrocardiogram (ECG) recordings during a 1-min rest period. HF-HRV was sampled from 0.12 to 0.40 Hz using spectral analyses of the ECG. Studies have shown this phenomenon to represent parasympathetic nervous system regulation and associations [37]. 

### Data analysis plan

Student’s t-tests and chi-squared analysis were performed to determine any differences in HR, PTSS, or violence exposure between race, sex, and site groups. Bivariate correlations were used to test associations among HR, HF-HRV, PTSS, and violence exposure. Moderation analyses were performed to test any direct or indirect effects of sex or resting HR on significant correlations. All analyses were performed in R.

## Results

### Participant characteristics

On average, children experienced ten different types of violent events (*M* = 10.29, *SD =* 5.97; see Table 1). There were no significant differences in violence exposure between participants grouped by sex, race, or site. Post-traumatic stress symptoms (PTSS) were assessed only in children who reported experiencing a criterion A traumatic event (*n* = 75). Among the 75 children who completed the UCLA-RI, average total PTSS severity was below the diagnostic criteria of 35 for partial PTSD (*M* = 23.81, *SD* = 14.80) [34, 38]. PTSS did not differ by sex or race, however the Detroit sample had significantly higher rates of PTSS compared to the Atlanta sample (Detroit, MI: *M* = 26.39, *SD =* 14.45 v. Atlanta, GA: *M* = 17.19, *SD* = 13.88, t = -2.55, *p* < 0.05). Resting HR of the sample averaged 83 beats per minute (BPM), (*M =* 83.07, *SD* = 10.79) and did not significantly differ by sex, race, or site. Girls reported greater pubertal development compared to boys, (Boys: *M = 5.42, SD = 1.83* v. Girls: *M = 6.61, SD = 1.85;* t = -3.12 *p* < 0.01).

### Association between violence exposure, posttraumatic stress symptoms, and resting heart rate

As expected, violence exposure was positively associated with PTSS, r(73) = 0.56, *p* < 0.01. The total number of types of violence exposure was negatively associated with resting HR, r(90) = -0.34, *p* < 0.01, as was PTSS, r(73) = -0.24, *p* = 0.04. On the other hand, level of violence exposure was positively associated with resting HF-HRV, r(81) = 0.26, *p* = 0.02; however PTSS was not associated with HF-HRV.

### Sex-specific analysis

**Fig. 1:**
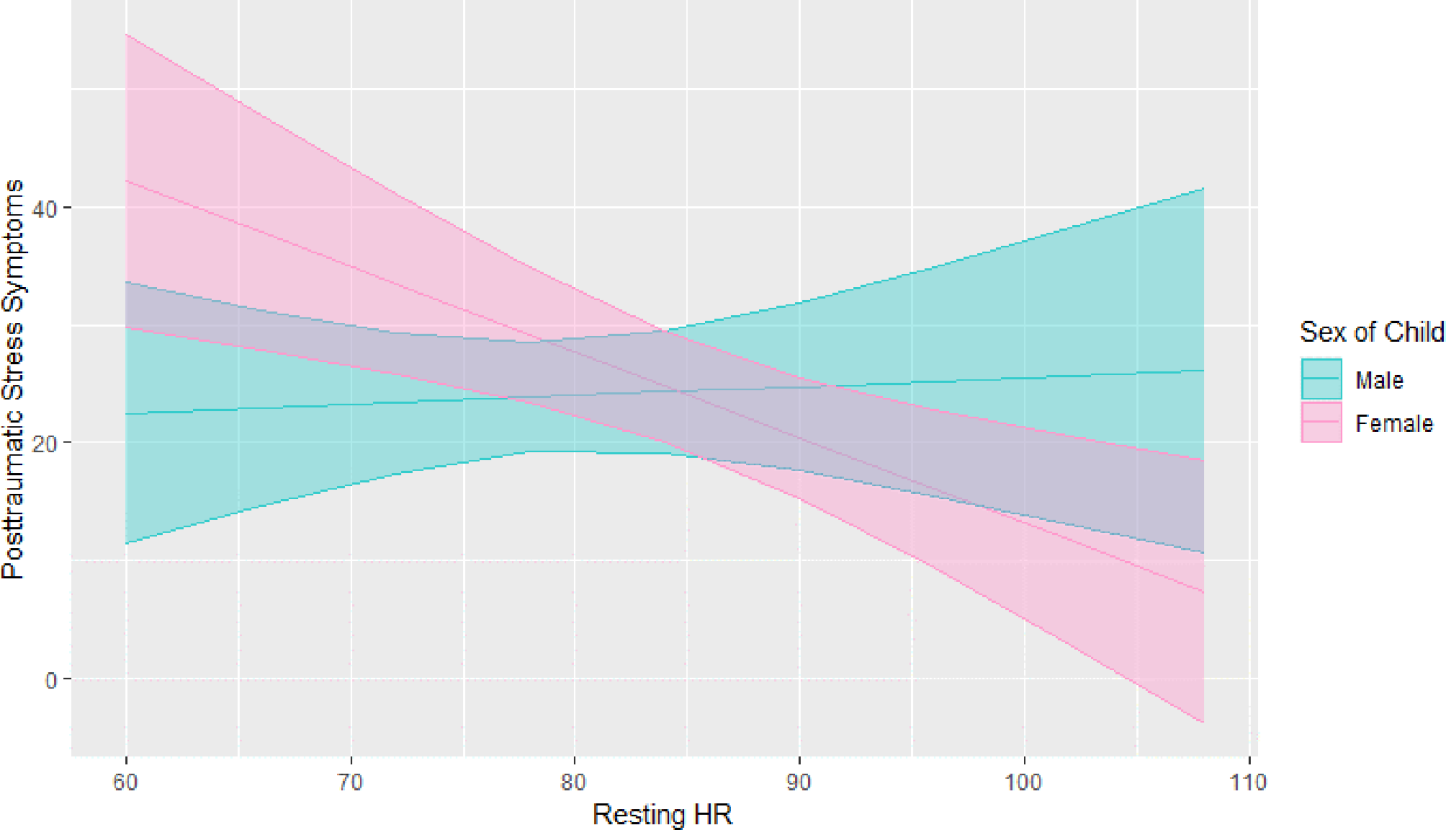
Estimated marginal means of PTSS by resting HR in boys and girls from our unadjusted moderation model, indicating sex of child significantly moderates the relationship between resting HR and PTSS, (interaction term: B = -0.80374, *p* = 0.023). In boys, resting HR was not found to be associated with PTSS, (r(36) = 0.05, *p* = 0.77), while girls’ lower resting HR was associated with higher PTSS, (r(35) = -0.47, *p* < 0.01) *Model*Y(PTSS) = β _0_ + β _1_ (resting HR) + β _2_ (sex) + β_3_ (PDS) + β _4_ (Violence exposure) + β _5_ (site) + β _6_ (sex * resting HR)

To account for differences in the pubertal progression of boys and girls at the age of our sample, sex was examined as a moderator between violence exposure and resting HR controlling for PDS. The interaction term between violence exposure and sex of child was not significant, *B* = -0.12, *p* = 0.77. Sex was also examined as a moderator affecting the association between resting HR and PTSS, controlling for PDS, violence exposure, and research site. The resulting interaction term between sex of child and resting HR was significant, (*B* = -0.64, *p* < 0.05), and a visual depiction of these results can be seen in Fig. 1. We also examined sex as a moderator in the HF-HRV analyses, but did not find significant interactions. Supplemental Table 1 shows the results of these analyses.

Given these results, we tested the relationships between violence exposure, HR, and PTSS by stratifying our analyses based on sex. As in the total sample, violence exposure was associated with PTSS in both boys, r (36) = 0.54, *p* < 0.01, and girls, r(35) = 0.58, *p* < 0.01. In boys, the correlation between violence exposure and resting HR trended toward significance, r(46) -0.28, *p* = 0.05. Girls’ violence exposure was significantly associated with lower resting HR, (r(39) = -0.40, *p* < 0.001). In boys, resting HR was not found to be associated with PTSS, (r(36) = 0.05, *p* = 0.77). In contrast, girls’ lower resting HR was associated with higher PTSS, (r(35) = -0.47, *p* < 0.01).

### Resting HR group

To further explore if sex significantly interacted with cardiovascular psychophysiology given the significant moderation effect, we performed exploratory analysis with standard clinical cutoffs of resting HR to determine if children who are below that cutoff might be at higher risk given trauma exposure. Participants’ resting HR was compared to age-appropriate normative ranges, as defined by the CDC. Participants were stratified into “normative resting HR” or “lower-than-normative resting HR” groups. It should be noted that the boys in our sample had a lower resting HR compared to the national average, t = 2.92, *p  < 0.05, Cohen’s D = 0.33.* There was not a significant difference in our sample of girls compared to the national average.

**Fig. 2:**
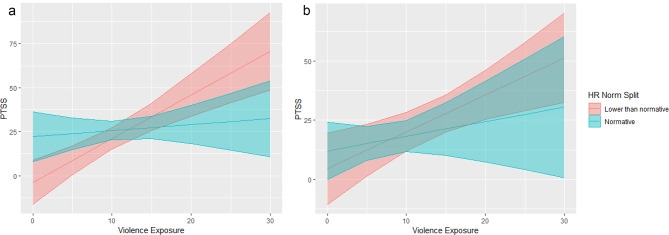
(**a**) Estimated marginal means depicting the moderating relationship of resting HR group on violence exposure and PTSS in boys. (**b**) Estimated marginal means depicting the relationship between violence exposure and PTSS by resting HR group

We found no significant differences in violence exposure, PTSS, or proportion of girls and boys by HR group. We tested to see if resting HR group moderated the relationship between violence exposure and PTSS and after controlling for covariates, found the effect of the interaction term was in the negative direction though not significant, (B = -0.95, *p* = 0.07), such that only those with below normative HR had a significant positive association between violence exposure and PTSS controlling for covariates. In line with our analysis plan, the moderation was stratified by sex to see how the association might differ in boys and girls. The normative HR group was found to significantly moderate the relationship between violence exposure and PTSS in boys, (B = -2.14, *p* < 0.01), but not girls (B = -0.94, *p* = 0.27). A visual representation of this association can be seen in Fig. 2a & b. The associated correlation coefficients between violence exposure and PTSS controlling for PDS and research site are as follows: boys and girls with lower than normative resting HR had a significant adjusted association, (r(14) = 0.78, *p* < 0.05; r(16) = 0.48, *p =* 0.02 respectively), whereas boys and girls with normative resting HR did not, (r(22) = 0.26, *p =* 0.05; r(19) = 0.67, *p* = 0.10, respectively). These findings suggest that resting HR may play a more important risk-identifying role in boys, such that boys with lower than normative resting HR show a significant association between violence exposure and PTSS while boys with normative resting HR show a blunted effect. In girls, it appears that the association between violence exposure and PTSS is positive, regardless of which HR group they belong to.

## Discussion

In our majority Black, urban children sample, we found that (1) violence exposure was associated with slower HR, (2) girls, but not boys showed greater PTSS associated with slower HR, and (3) boys with lower than normative HR showed a stronger association between violence exposure and PTSS compared to boys with normative HR. Our results replicate prior findings that increased violence exposure in Black youth is associated with increased PTSS and slower HR [20]. In the previously cited study, slower HR was shown to be linked with signs of accelerated epigenetic aging suggesting that violence exposure might be associated with a more adult-like HR. Such accelerated development is consistent with studies in children with early adversity showing increased maturation in brain and behavior, and could be a marker of risk for future negative health outcomes [40]. Our sample’s demonstration of slower more adult-like resting HR being associated with higher violence exposure could provide an important prior step to Hao et al’s 2022 analysis of resting HR trajectories and left ventricular mass in adulthood, such that our study could give insight into why some children’s resting HR decreases more quickly than others [14]. 

With respect to sex differences, we found that sex moderated the association between resting HR and PTSS, such that girls showed a significant negative association and boys showed no association. Sex differences in autonomic functioning and their relation to PTSD symptom severity have been previously demonstrated in adults, however our analysis in pre-adolescent children gives evidence of this phenomenon earlier in development [41]. One potential mechanism driving these sex difference at such an early age could include pubertal timing in response to early life stress, although findings are mixed [27]. In addition, the HF-HRV analyses suggest that there may be over-regulation by the PNS in girls with high levels of violence exposure.

Our stratification by normative resting HR group was in effort to provide clinical significance to a psychophysiological measure in that resting HR below the clinical cutoff may serve as an additional data point for providers when assessing well-being. For example, it could be used as a part of a screener, alerting providers to the need for a more comprehensive assessment. Resting HR, and the extent to which it differs from the clinical cutoff, may be integrated with patients’ psychosocial histories to provide a holistic picture of the patient, grounded in a biopsychosocial model of well-being. It could also serve as a predictive biomarker for mental health risk as the body of research surrounding the topic grows. Recent studies have explored resting HR during adolescence as a predictive measure for later hypertension or have explored models of resting HR trajectory in association with other risk factors, but ours is the first to explore this potential biomarker in the context of development and mental health [39, 42]. In addition, our sample’s demonstration of psychological consequences in addition to the physiological implications could provide new information about a psychobiological sequelae of violence exposure.

### Perspectives and significance

Our findings add to the body of evidence that childhood violence exposure has a negative impact on cardiovascular functioning and point to important new information about sex-specific risk factors [14]. The slower, more adult-like resting HR coupled with the increase in PTSS in girls could indicate both physiological and psychological consequences of violence.

It should be noted that a limitation of the study was that these data were collected cross-sectionally and although our exposure was retrospectively recorded, only non-causal associations can be interpreted. Similarly, our use of self-report measures could introduce recall bias leading to a mismeasurement of violence exposure; however, collecting the data from 9-year-old children limits the problems with adults retrospectively reporting on their children’s history. In addition, our use of child self-report measures also represents a strength in the validity and accuracy compared to relying on caregiver report of child’s experiences. Lastly, our primarily Black, urban sample gives attention to historically underrepresented populations in research while enhancing the generalizability of the findings.

## Conclusions

Violence exposure was found to negatively impact children’s physiology through the association with lower resting heart rate. Girls and boys showed differential patterns of PTSS related to this physiological alteration. Future studies should examine potential mechanisms underlying this sex difference to best support at risk children and their unique challenges. Specifically, longitudinal studies may be able to help researchers understand how reduced HR during childhood might lead to future cardiovascular disease and psychopathology.

**Table 1:** Participant characteristics

	Male (*N*=48)	Female (*N*=44)	Total (*N*=92)
**Age in Years**			
Mean (SD)	9.43 (0.336)	9.45 (0.407)	9.44 (0.370)
**Pubertal Development Scale Scum**			
Mean (SD)	5.42 (1.83)	6.61 (1.85)	5.99 (1.92)
**Race**			
Mixed Ancestry	12 (25.0%)	4 (9.1%)	16 (17.4%)
African American/Black	36 (75.0%)	40 (90.9%)	76 (82.6%)
**Research Site**			
Atlanta, GA	14 (29.2%)	12 (27.3%)	26 (28.3%)
Detroit, MI	34 (70.8%)	32 (72.7%)	66 (71.7%)
**Resting HR**			
Mean (SD)	81.6 (10.8)	84.7 (10.6)	83.1 (10.8)
**Resting HR Norms**			
Below Normative	17 (35.4%)	21 (47.7%)	38 (41.3%)
Normative	31 (64.6%)	23 (52.3%)	54 (58.7%)
**Violence Exposure**			
Mean (SD)	10.7 (5.84)	9.86 (6.14)	10.3 (5.97)
**PTSS Severity**			
Mean (SD)	23.9 (13.8)	23.7 (15.9)	23.8 (14.8)

### Electronic supplementary material

Below is the link to the electronic supplementary material.

Supplementary Material 1

Supplementary Material 2

## Data Availability

The datasets used and/or analyzed during the current study are available from the corresponding author on reasonable request.
