# Supplementary material for: Resting heart rate associations with violence exposure and posttraumatic stress symptoms: sex differences in children
Source: Biol Sex Differ. 2024 Mar 28;15:28. doi: 10.1186/s13293-024-00606-2 (PMC10976797; doi:10.1186/s13293-024-00606-2)
Supplement: Supplementary file 1 — Supplementary Material 1 [file 13293_2024_606_MOESM1_ESM.docx]

Supplement

Given that lower resting HR may be driven by an over-regulation of the ANS, we looked at associations between variable of interest and resting high-frequency heart rate variability (HF-HRV).

Results

Supplement Table 1.

|  | Resting HF-HRV | Girls | Boys |
| --- | --- | --- | --- |
| Violence exposure  *Pearson correlation and p-value* | r(81) = 0.26,  *p* = 0.02 | r(37) = -.30,  *p* = 0.06 | r(42) = 0.21,  *p* = 0.18 |
| PTSS  *Pearson correlation and p-value* | r(68) = 0.08,  *p* = 0.53 | r(31) = 0.28,  *p* = 0.11 | r(35) = -0.16,  *p* = 0.36 |
| $Y\left( PTSS \right)=\beta_{0} + \beta_{1}(resting HF-HRV) + \beta_{2}(sex) + \beta_{3}(PDS)+ \beta_{4}(violence exposure)+ \beta_{5}(site)+ \beta_{6}(sex*resting HF-HRV)$  *Beta coefficient of the interaction term in the adjusted model and p-value*  *PDS = pubertal development scale* | $\beta_{6}(sex*resting HF-HRV)$= 4.98  *p* = 0.0543 | | |

Resting HR was negatively associated with violence exposure, such that the more violence exposure a child reported on average the lower their resting HR. The opposite effect was seen with relationship to resting HF-HRV, such that children with greater violence exposure had higher resting HF-HRV.

Lastly, when we explored whether sex moderated the relationship between resting HF-HRV and PTSS, we found a positive, trending association. The association between violence exposure and HF-HRV was stronger in girls than boys, but was still only at trend level.
