# Supplementary material for: Resting heart rate associations with violence exposure and posttraumatic stress symptoms: sex differences in children
Source: Biol Sex Differ. 2024 Mar 28;15:28. doi: 10.1186/s13293-024-00606-2 (PMC10976797; doi:10.1186/s13293-024-00606-2)
Supplement: Supplementary file 2 — Supplementary Material 2 [file 13293_2024_606_MOESM2_ESM.docx]

We thank the reviewers for their careful evaluation of the paper. As suggested by the reviewers, we have expanded the introduction and discussion, added references and additional analyses, modified a figure to be clearer, and added more details about participants. We believe the manuscript is much stronger; the revisions are highlighted in the manuscript. Below we address each reviewer concern:

Reviewer #1:

This manuscript by Wiltshire et al entitled "Resting Heart Rate Associations with Violence Exposure and Posttraumatic Stress Symptoms: Sex Differences in Children" delves into the previously studied association between violence exposure, post-traumatic stress symptoms, and heart rate, focusing on a sample mainly made up of black American children. Overall, I found this to be an interesting and promising manuscript, as it shines light on a very specific field, which has shown contradictory results up to now. Only a few minor aspects of it take away from the general work.

Major concerns
In the methods - participants section, it is stated that "There were no differences in demographic or clinical characteristics between participants included and excluded in this analysis", but no more information is provided. When looking at Table 1, no clinical variables are specified. Was this analysis of clinical characteristics done based on the lack of specific diagnoses, were clinical variables analyzed, and were previous psychological/cardiac evaluations used…?

Thank you for bringing this to our attention. We have added language to the Methods section, participants subheading, to clarify. We have now specified that: “The ten individuals excluded from the analysis did not differ in age or sex, nor did they differ in levels of PTSS or violence exposure compared to the 92 children included in our analysis.”

On page 11 of the document, the authors claim that after testing to see if the resting HR group moderated the relationship between violence exposure and PTSS and after controlling for covariates, they found the effect is in the direction they would expect, though not significant. Even after this sentence, it is not clear to me the direction this effect takes, as no hypotheses were made about HR cut-off results. In the hypothesis section, it is only specified that these exploratory analyses were performed.

Our response to this suggestion is two-fold. First, we added an additional citation that provides evidence that lower resting HR in childhood/adolescence is associated with negative cardiac risk factors in adulthood.

Guang Hao, James Halbert, Shayong Su, Zsolt Bagi, Vincent Robinson, Julian Thayer, Gregory Harshfield & Gaston Kapuku (2021) Rapid decline of resting heart rate trajectories from childhood to young adulthood is paradoxically associated with increased cardiac mass, Acta Cardiologica, 76:10, 1117-1123, DOI: 10.1080/00015385.2020.1871262

With this additional support from the literature, we have added a more specific prediction regarding our exploratory analysis.

“In addition, we performed exploratory analysis with participants categorized into normative versus blunted resting cardiac functioning around CDC recommended standardized ranges of resting HR for 9-year-old children to see if lower resting HR, relative to normative reference points, was associated with negative mental health outcomes.”

Second, we removed the language in our results regarding “the direction we would expect” and provide a more straightforward explanation.

“We tested to see if resting HR group moderated the relationship between violence exposure and PTSS and after controlling for covariates, found the effect of the interaction term was in the negative direction, though not significant, (B = -0.95, p = 0.07), such that only those with below normative HR had a significant positive association between violence exposure and PTSS controlling for covariates.”

In Figure 1 it seems as if there is an opposite relationship between PTSS and HR, but in the results section it's stated that "In boys, resting HR was not found to be associated with PTSS, (r(36) = 0.05, p = 0.77). In contrast, girls' lower resting HR was associated with higher PTSS, (r(35) = -0.47, p< 0.01)". I understand this figure is just illustrative, but It becomes confusing. I recommend either accompanying It with separate graphs for each gender, adding some mark that indicates the significance only in girls, or showing the real correlations (which based on the statistics would be more of a horizontal line for boys, and the downward heading line already in the figure for girls).

We have changed Figure 1 to better illustrate the specific results mentioned per your suggestion. Previously the figure showed a graph of the estimated marginal means from our generalized linear model with a significant interaction term, controlling for several covariates: PDS, violence exposure, and research site. The result we are trying to convey with this figure is that the relationship between resting HR and PTSS looks differently in boys and girls, such that girls show a negative relationship and boys show no relationship. While this is best interpreted from the significant interaction term in our model, it is best visualized through the sex stratified correlations in the raw data. The new Figure 1 (included below) now demonstrates the estimated marginal means of PTSS by resting HR in boys and girls from our unadjusted moderation model, indicating sex of child significantly moderates the relationship between resting HR and PTSS.

Figure 1. NEW Estimated marginal means of PTSS by resting HR in boys and girls from our unadjusted moderation model, indicating sex of child significantly moderates the relationship between resting HR and PTSS, (interaction term: B = -0.80374, p = 0.023). In boys, resting HR was not found to be associated with PTSS, (r(36) = 0.05, p = 0.77), while girls’ lower resting HR was associated with higher PTSS, (r(35) = -0.47, p< 0.01).

Figure 1.OLD Line graph indicating sex of child significantly moderates the relationship between resting HR and PTSS controlling for PDS, violence exposure, and research site.

Reviewer #2:

1. This is a novel and impactful paper, and I appreciated the opportunity to review it. The understudied and high risk sample, as well as the examination of sex differences are major strengths. I have minor comments for consideration.

2. Given that higher regulation among the girls (due to higher PTSS) may explain lower HR, did the authors look at HRV/RSA? Presumably one of the limitations to the prior literature is that authors haven't looked at whether higher HRV/RSA accounts for the lower HR findings, so if the authors could show that, it would really increase the impact. If it doesn't explain the findings, perhaps this suggests that there is another mechanism leading to lower HR.

This is a very good point. One of our primary goals of this paper was to follow-up on our previous work in children with trauma exposure (Jovanovic et al, 2017) in which we found that violence was associated with lower HR but not HRV. Although we want to keep the focus of the current paper on HR, we added new analyses of HF-HRV, and found the opposite direction of effects with violence exposure (i.e. positive association with resting HF-HRV), although there was only a trend for an interaction with sex, and no association with PTSS (see table below). These results suggest that the lower resting HR we observed might indeed be driven by over-regulation by the PNS. We have added these analyses to the results section and have added the point to the discussion. More detailed analyses of HF-HRV are provided in the Supplement.

|  | Resting HR | Resting HRV |
| --- | --- | --- |
| Violence exposure | r(90) = -0.34, p < 0.01 | r(81) = 0.26,  *p* = 0.017 |
| PTSS | r(73) = -0.24,  p = 0.04 | r(68) = 0.077,  *p* = 0.53 |
| PTSS = restingH + sex + PDS + violence exposure + Site + sex*resting | *B(sex*resting HR)* = -0.64,  *p* < 0.05 | B(sex*resting HRV) = 4.98  *p* = 0.0543 |

3. In the Introduction, it would be helpful if the authors could explain why lowered HR might be problematic from a cardiovascular standpoint and from a mental health outcomes standpoint. This gets back to the point about HRV/RSA - if these children are over-regulating, how may that end up being problematic?

We added an additional citation that provides evidence that lower resting HR in childhood/adolescence is associated with negative cardiac risk factors in adulthood.

Guang Hao, James Halbert, Shayong Su, Zsolt Bagi, Vincent Robinson, Julian Thayer, Gregory Harshfield & Gaston Kapuku (2021) Rapid decline of resting heart rate trajectories from childhood to young adulthood is paradoxically associated with increased cardiac mass, Acta Cardiologica, 76:10, 1117-1123, DOI: 10.1080/00015385.2020.1871262

4. The authors did not find significant differences in PTSS by sex or race - this seems to contradict some of the prior literature, which should be discussed.

Much of the previous literature has found sex and racial differences in PTSD in adult samples. PTSD symptoms are only just presenting at this age indicating that most of the sex differences would emerge in late adolescence. However, heart rate might be an early risk factor that’s predictive of future sex differences in PTSD. In addition, while our primarily Black sample of children provides important exposure to an understudied population, we unfortunately are not powered to find racial differences.

5. In the Discussion, this sentence should be tempered as I would not say that these associations are well-established: "Sex differences in autonomic functioning and their relation to PTSD symptom severity have been well established."

We have tempered the language in the discussion:

“Sex differences in autonomic functioning and their relation to PTSD symptom severity have been previously demonstrated in adults, however our analysis in pre-adolescent children gives evidence of this phenomenon earlier in development.”

6. The authors state, "Our findings add to the body of evidence that childhood violence exposure has a negative impact on cardiovascular functioning." Similar to my third point, there needs to be an explanation of how lowered HR is problematic for cardiovascular functioning.

We added an additional citation that provides evidence that lower resting HR in childhood/adolescence is associated with negative cardiac risk factors in adulthood.

Guang Hao, James Halbert, Shayong Su, Zsolt Bagi, Vincent Robinson, Julian Thayer, Gregory Harshfield & Gaston Kapuku (2021) Rapid decline of resting heart rate trajectories from childhood to young adulthood is paradoxically associated with increased cardiac mass, Acta Cardiologica, 76:10, 1117-1123, DOI: 10.1080/00015385.2020.1871262

7. The use of the clinical HR cutoff is interesting - it would be helpful if the authors could say a little more about its potential clinical utility.

We have added an additional statement in the discussion that describes how HR cutoff can be used to provide important context regarding the patient or its use as a biomarker.

“Our stratification by normative resting HR group was in effort to provide clinical significance to a psychophysiological measure in that resting HR below the clinical cutoff may serve as an additional data point for providers when assessing well-being. For example, it could be used as part of a screener, alerting providers to the need for a more comprehensive assessment. Resting HR, and the extent to which it differs from the clinical cutoff, may be integrated with patients’ psychosocial histories to provide a holistic picture of the patient, grounded in a biopsychosocial model of well-being. It could also serve as a predictive biomarker for mental health risk as the body of research surrounding the topic grows.”
